# Editorial: The Green Side of the Water Cycle: New Advances in the Study of Plant Water Dynamics

**DOI:** 10.3389/fpls.2020.582846

**Published:** 2020-09-02

**Authors:** Juan Pedro Ferrio, Maren Dubbert, Cristina Máguas

**Affiliations:** ^1^Aragon Agency for Research and Development (ARAID), Zaragoza, Spain; ^2^Department of Forest Resources, Agrifood Research and Technology Centre of Aragon (CITA), Zaragoza, Spain; ^3^Ecosystem Physiology, University of Freiburg, Freiburg, Germany; ^4^Landscape Ecohydrology, IGB Berlin, Berlin, Germany; ^5^Centre for Ecology, Evolution and Environmental Changes (cE3c), Faculdade de Ciências da Universidade de Lisboa, Lisboa, Portugal

**Keywords:** water uptake, water transport, hydrological tracers, water status, stable isotopes, noninvasive techniques, plant-fungi interactions, water footprint

Dynamics within the water cycle is a crucial topic under current climate change conditions. To understand terrestrial ecosystems and their role in the water cycle, we need to characterize the uptake, storage and transport of water along the soil-plant-atmosphere continuum. Assessing these processes is still challenging due to the trade-off between the uncertainty of upscaling approaches and the complexity of *in situ* direct measurements. In this Research Topic, we integrated the information available from multiple disciplines, from plant hydraulics to economy, going through material science and biomolecular techniques, covering a wide range of temporal and spatial scales.

Stable isotope analysis is a long-used powerful tool to study plant water relations. However, the temporal resolution of stable isotope analysis based on classical destructive sampling techniques is limited, precluding the characterization of highly dynamic hydrological processes. Here, two articles focused on methods for continuous monitoring of the isotopic signature of water, either in the soil (Kübert et al.) or in the tree stem (Marshall et al.). Kübert et al. assessed the use of a gas-permeable membrane to monitor soil water vapor by isotope ratio infrared spectrometry (IRIS). Compared to standard methods, in-situ soil water isotopic monitoring showed similar results, and tracked fast changes in soil water, although it might be affected by spatial heterogeneity, and the alteration of soil structure during installation. Marshall et al. tested a method in which air flows through a borehole in the tree trunk, measuring water vapor (equilibrated with stem water) by IRIS. The article shows that the method detects fast changes in source water, but faces some potential limitations, e.g., in species with narrow active sapwood, or small stems.

Also aimed at in-field continuous monitoring, Scalisi et al. and Scalisi et al. shifted the usual focus on leaves and stems toward the assessment of fruits. Changes in fruit (nectarine and olive) diameter and leaf pressure were monitored together with standard methods for determining plant water status. In a study on nectarine under deficit irrigation, Scalisi et al. showed how nocturnal fruit growth peaked at intermediate water potentials (*ca*. -1.5 MPa), but declined rapidly under lower water potentials, highlighting the suitability of the method to determine optimal irrigation regimes. In the case of olive trees, Scalisi et al. found that different cultivars may prioritize either leaf or fruit water status, suggesting that the comparison of one single compartment may lead to wrong conclusions regarding tree water status.

At the leaf level, Gomez Alvarez-Arenas et al. presented a contactless method, based on ultrasonic resonance, which provides information on different leaf properties, such as thickness and density. In this work, the method allowed to determine the surface density of the palisade and spongy mesophyll in three species (*Ligustrum lucidum*, *Vitis vinifera*, and *Viburnum tinus*). The technique showed, in a non-invasive way, the relative contribution of palisade and spongy mesophylls to leaf thickness, as well as the differential responses of these two layers to leaf water loss.

Two articles focused on the regulation of plant water transport. In an experimental study with maize, Hayat et al. tracked the changes in transpiration and water potential during soil drying, in order to assess whether stomata are downregulated to minimize losses in hydraulic conductance. Comparing root-pressurized and non-pressurized plants, they concluded that losses in soil-plant hydraulic conductance drove stomatal closure in maize. At the other extreme of the soil-plant-atmosphere continuum, Du et al. assessed the acclimation to high vapor pressure deficit (VPD) of two tomato cultivars. The cultivar Jinpeng showed an anisohydric behavior, with high VPD inducing an increase in whole-plant hydraulic conductance, keeping high stomatal conductance and transpiration rates. Conversely, the cultivar Zhongza displayed a more conservative, isohydric strategy, showing a coordinated decline in stomatal and hydraulic conductance. Interestingly, the contrasting acclimation strategies involved differential changes in anatomical traits.

In connection with the study of water transport, Xu and Zwiazek presented in a *Perspectives* paper a theoretical framework on the regulation of plant water status through the aquaporins of ectomycorrhizal fungi, discussing existing evidence and future challenges. Although a moderate increase in fungal aquaporin expression may enhance root hydraulic conductivity, under adverse conditions it may cause opposite effects. In this context, the authors highlighted the need to assess the coordination between plant and fungal aquaporins under water stress.

Finally, the *Opinion* paper by Cazcarro and Bielsa addressed, from an economic viewpoint, current issues in the quantification of water footprints at regional scales. Firstly, they highlighted the lack of consensus regarding what should be considered as anthropogenic water footprint, exemplified by the case of forestry. They also discussed the challenges in upscaling transpiration and evaporation to regional scales, concluding with some recommendations on how to improve the interaction between natural and social sciences in the valorization of water resources.

Although far from a comprehensive compilation of methods, we gathered new conceptual approaches and applications, with a particular focus on continuous-monitoring methods. Bearing in mind the highly dynamic nature of soil-plant-atmosphere interactions, the techniques presented here are likely to foster our knowledge on the mechanism and regulation of plant water use. Besides the application to plant ecophysiology and precision agriculture, online sensing of the “green side” of the water cycle is needed to fine-tune ecohydrological models, e.g., to anticipate plant responses to climate change, and their implications for land water resources.

## Author Contributions

JF prepared the first draft of this editorial. MD and CM revised the editorial. All authors contributed to the article and approved the submitted version.

## Funding

JF was supported by Reference Group H09_20R (Gobierno de Aragón, Spain) and the project PID2019-106701RR-I00/AEI/10.13039/501100011033. CM acknowledges financial support from Fundação para a Ciência e Tecnologia (FCT), through the strategic project UIDB/00329/2020 granted to the Centre for Ecology, Evolution and Environmental Changes, cE3c, Faculdade de Ciências, Universidade de Lisboa. MD was supported by the German Science Foundation (DFG; DU1688/1-1, DU1688/4-1).

## Conflict of Interest

The authors declare that the research was conducted in the absence of any commercial or financial relationships that could be construed as a potential conflict of interest.

